# Control energy of complex networks towards distinct mixture states

**DOI:** 10.1038/s41598-018-29207-x

**Published:** 2018-07-18

**Authors:** Sen Nie, H. Eugene Stanley, Shi-Ming Chen, Bing-Hong Wang, Xu-Wen Wang

**Affiliations:** 1 School of Electrical and Automation Engineering, East China Jiaotong University, Nanchang, Jiangxi 330013 China; 2 Department of Physics and Center for Polymer Studies, Boston University, Boston, MA 02215 USA; 3 Department of Modern Physics, University of Science and Technology of China, Hefei, Anhui 230026 China

## Abstract

Controlling complex networked systems is a real-world puzzle that remains largely unsolved. Despite recent progress in understanding the structural characteristics of network control energy, target state and system dynamics have not been explored. We examine how varying the final state mixture affects the control energy of canonical and conformity-incorporated dynamical systems. We find that the control energy required to drive a network to an identical final state is lower than that required to arrive a non-identical final state. We also demonstrate that it is easier to achieve full control in a conformity-based dynamical network. Finally we determine the optimal control strategy in terms of the network hierarchical structure. Our work offers a realistic understanding of the control energy within the final state mixture and sheds light on controlling complex systems.

## Introduction

Because it can accurately characterize such real-world systems as social networks^1–3^, biological networks^4–7^, technical networks^8,9^, and financial networks^10–13^, network science has been a popular research topic for decades. In recent years in particular, various ways of controlling them have been devised^14–20^, which aims to figure out whether external inputs can be used to drive a networked system from an initial state to any desired final state within a finite period of time^21^. Controllability, i.e., the minimum number of inputs (driver nodes) required to achieve the full control, has been examined theoretically both from the structural controllability of directed networks with random link weights^22^ and of networks with arbitrary structures and link weights^23^.

When achieving control, it is expected to minimize the control energy required to steer a system from any arbitrary initial state to a desired final state. The control energy relies strongly on the controllability Gramian^24–29^, which includes topological properties, control time, initial and final states, and the number of inputs. It has been proved that the time regime and degree correlation can confine the lower and upper bounds of the control energy^26^. When exploring the eigenspace of the controlled system, we find that the eigen-energies can be either heterogeneous or homogeneous, depending on how we control the different node fractions^27^.

Although prior works have focused on the selection of driver nodes that can reduce the control energy^30–32^, the goal when controlling a networked system is usually to steer each node towards an identical state. For example, an entire swarm of honeybees can be drawn into the same nest only by a few “shepherding” honeybees^33^. Because synchronization—where all individuals reach a consistent phase—is prevalent in nature^34–36^, we need to know whether it requires more control energy to realize this identical final state than a non-identical mixed final state. In addition, nodes tend to tune their states to synchronize with those of their network neighbors. This conformity behavior is prevalent in both natural and social systems^37,38^. Since conformity behavior-based dynamics facilitates the controllability of an identical state^39^, we examine the control energy of such a conformity-based dynamical network.

Here, we explore the control energy by examining the number of driver nodes needed to direct the system from an initial state to either an identical or non-identical final state. We incorporate conformity dynamics into the general model to determine how nodal dynamics affect the control energy. Using simulations of synthetic and real networks, we find the relationship between the minimal driver nodes and control energy and determine the optimal set of driver nodes for minimizing the control energy.

## Results

### Canonical linear model

We use a dynamical system governed by the canonical linear equation (see Method) for modeled Erd*ö*s-Rényi^40^ and scale-free^41^ networks to determine the control energy required to steer the system from the initial state **x**_o_ to the following final state with different mixtures by choosing all of the nodes as driver nodes: (i) an identical final state ${{\bf{x}}}_{{\rm{f}}}^{({\rm{ID}})}$, where the final state of each node *x*_*i*_(*t*_f_) is a constant *c*, (ii) a non-identical final state ${{\bf{x}}}_{{\rm{f}}}^{({\rm{NI1}})}$, where the final state of each node is drawn from the uniform distribution ${x}_{i}({t}_{{\rm{f}}})\sim {\bf{U}}(0,c)$, and (iii) a non-identical final state ${{\bf{x}}}_{{\rm{f}}}^{({\rm{NI2}})}$, where the final state of each node is drawn from the uniform distribution ${{\bf{x}}}_{i}({t}_{{\rm{f}}})\sim {\bf{U}}(0,\sqrt{3}c)$. This setup guarantees the fairness of comparison between ${{\bf{x}}}_{{\rm{f}}}^{({\rm{ID}})}$ and ${{\bf{x}}}_{{\rm{f}}}^{({\rm{NI2}})}$ because their norms are the same.

**Figure 1:**
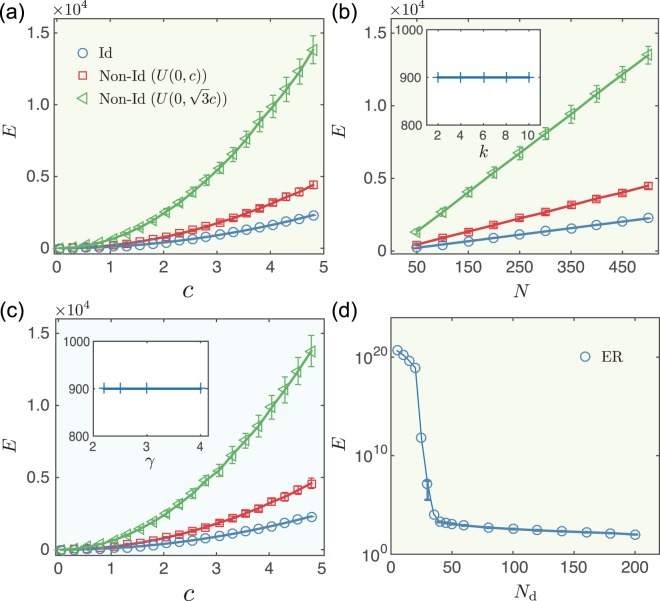
Control energy for undirected networks with identical (Id) and non-identical (Non-Id) final state modes. (**a**) Control energy *E* as a function of distance *c* in directing the random network to distinct final state modes. Network size *N* = 200 and the average degree *k* = 5. (**b**) Control energy *E* as a function of network sizes *N* in directing the random network to distinct final state modes. Inset: Control energy as a function of average degrees *k* in directing the random network to the identical final state (*c* = 3). (**c**) Control energy *E* as a function of distance *c* in directing the scale-free network to distinct final state modes (*γ* = 2.5, *k* = 5). Inset: Control energy as a function of power exponents *γ* in directing the scale-free network to the identical final state (*k* = 5, *c* = 3). (**d**) Control energy *E* as a function of the number of driver nodes *N*_d_ in directing the random network to the identical final state (*c* = 1). The link weights of all networks are uniformly drawn from *U*(0, 1). The error bars represent standard deviations and each data point is an average over 100 independent realizations.

Figure 1(a) shows there are no prominent differences among the control energies of the identical mode *E*_ID_ and the two non-identical modes *E*_NI1_ and *E*_NI2_ for small *c*, i.e., the final state is not far from the initial state. However, this energy gap expands with parameter *c*, indicating that it is easier to direct the networked system to an identical final state than to non-identical ones if the control distance is greater (see Fig. 1(a)).

**Figure 2:**
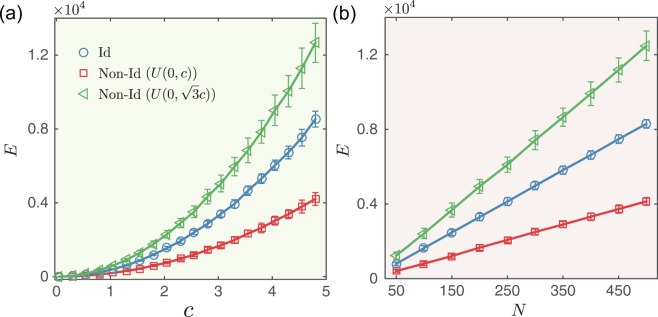
Control energy for directed random networks with identical (Id) and non-identical (Non-Id) final state modes. (**a**) Control energy *E* as a function of distance *c*. Network size *N* = 200 and average degree *k* = 5. (**b**) Control energy *E* as a function of network size *N* (*c* = 3). The error bars represent standard deviations and each data point is an average over 100 independent realizations.

For a specific final state mode, i.e., *c* = 3, the control energy *E* is linearly dependent on network size *N*, regardless of whether ${{\bf{x}}}_{{\rm{f}}}^{({\rm{ID}})}$, ${{\bf{x}}}_{{\rm{f}}}^{({\rm{NI1}})}$ or ${{\bf{x}}}_{{\rm{f}}}^{({\rm{NI2}})}$ is considered. Although we expect that the control energy in networks with different link densities (as in the random networks) and with different power exponents (as in the scale-free networks) to differ, after all, the controllability Gramian matrix $G({t}_{{\rm{f}}})={\int }_{0}^{{t}_{{\rm{f}}}}{{\rm{e}}}^{At}B{B}^{{\rm{T}}}{{\rm{e}}}^{{A}^{{\rm{T}}}t}{\rm{d}}t$ includes the term of the coupling matrix *A* (see Method), but the control energy *E* for an identical final state mode is virtually independent with the average degree and power exponent (see inset of Fig. 1(b,c)). Figure 1(d) in particular shows that a slight increase in the number of inputs dramatically reduces the control energy needed to reach an identical final state. Thus network size is the significant contributor to the control energy of a networked system achieving identical mode and not the average degree or the power exponent. We also examine the control energies *E*_ID_, *E*_NI1_ and *E*_NI2_ for directed random networks, and generally find that *E*_NI2_ is higher than *E*_ID_, whereas *E*_NI1_ is lower than *E*_ID_ (see Fig. 2(a)).

**Figure 3:**
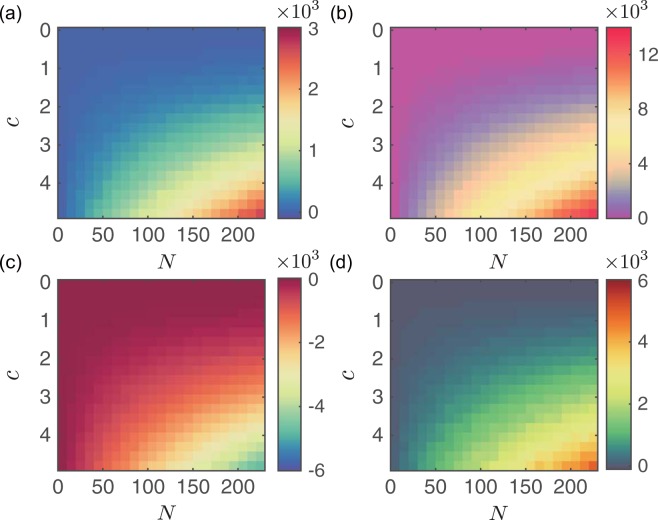
Energy gap between the non-identical and identical final state modes for random networks. (**a**) Energy gap Δ*E*_1_ ≡ *E*_NI1_ − *E*_ID_ as functions of network size *N* and distance *c* for undirected random networks. (**b**) Energy gap Δ*E*_2_ ≡ *E*_NI2_ − *E*_ID_ as functions of network size *N* and distance *c* for undirected random networks. (**c**) Energy gap Δ*E*_1_ as functions of network size *N* and distance *c* for directed random networks. (**d**) Energy gap Δ*E*_2_ as functions of network size *N* and distance *c* for directed random networks. The average degree *k* = 5. Each data point is an average over 100 independent realizations.

To systematically examine the energy gap between final states with different mixtures, we compare Δ*E*_1_ ≡ *E*_NI1_ − *E*_ID_ and Δ*E*_2_ ≡ *E*_NI2_ − *E*_ID_ on the *N*−*c* plane. Figure 3(a,b) show the results in undirected networks. Note that (i) *E*_NI1_  < *E*_ID_ for small network size *N* and short distance *c*, and (ii) ${E}_{{\rm{NI1}}} > {E}_{{\rm{ID}}}$ as *N* and *c* exceed the critical values. Figure 3(c,d) show the results in directed networks, in which we find that *E*_NI1_ < *E*_ID_ for all combinations of *N* and *c* in directed networks, and that *E*_NI2_ is always higher than *E*_ID_ in both undirected and directed networks.

To heuristically explain why *E*_ID_ is generally smaller than *E*_NI_, we examine a simple networked system with two nodes and assume the inverse of the Gramian matrix *G*^−1^(*t*_f_) to be

1 $${G}^{-1}({t}_{{\rm{f}}})=[\begin{array}{cc}{g}_{11} & {g}_{12}\\ {g}_{21} & {g}_{22}\end{array}]\mathrm{.}$$

Note that in undirected networks *g*_12_ = *g*_21_. We denote the final state of non-identical modes as ${{\bf{x}}}_{{\rm{f}}}^{({\rm{NI}})}={[{x}_{{\rm{f1}}},{x}_{{\rm{f2}}}]}^{{\rm{T}}}$, and the final state of identical mode to be ${{\bf{x}}}_{{\rm{f}}}^{({\rm{ID}})}={[c,c]}^{{\rm{T}}}$. Because the final state of non-identical mode is drawn from uniform distribution **U**(0, *c*), we assume *x*_f1_ < *x*_f2_ = *c* (Note that this can be extended to the case ${x}_{{\rm{f1}}} > {x}_{{\rm{f2}}}$). The control energy *E* required to direct a networked system from an initial state to a final state is (see Method for details)

2 $$E({t}_{{\rm{f}}})={{\bf{x}}}_{{\rm{f}}}^{{\rm{T}}}{G}^{-1}({t}_{{\rm{f}}}){{\bf{x}}}_{{\rm{f}}}\mathrm{.}$$

Substituting the final states into Eq. (2), we obtain the control energies *E*_NI_ and *E*_ID_

3 $$\begin{array}{rcl}{E}_{{\rm{NI}}} & = & {{x}_{{\rm{f1}}}}^{2}{g}_{11}+{{x}_{{\rm{f2}}}}^{2}{g}_{22}+{x}_{{\rm{f1}}}{x}_{{\rm{f2}}}{g}_{21}+{x}_{{\rm{f1}}}{x}_{{\rm{f2}}}{g}_{12},\\ {E}_{{\rm{ID}}} & = & {c}^{2}{g}_{11}+{c}^{2}{g}_{22}+{c}^{2}{g}_{21}+{c}^{2}{g}_{12}\mathrm{.}\end{array}$$

The energy gap between two modes is

4 $$\begin{array}{l}{\rm{\Delta }}E=({{x}_{{\rm{f1}}}}^{2}-{c}^{2}){g}_{11}+({x}_{{\rm{f1}}}c-{c}^{2})({g}_{21}+{g}_{12}\mathrm{).}\end{array}$$

Equation. (4) indicates that Δ*E* is supported by two terms, one determined by node 1—which is reasonable because the final state of node 2 is the same for two modes— and a second that is the coupling effect between node 1 and node 2. From Eq. (4) we conclude:

(i) That *g*_21_ = *g*_12_ = 0, i.e., there is no association between nodes 1 and 2. For any $c > 0$, Eq. (4) yields Δ*E* < 0, indicating that it is easier to control this system towards a non-identical final state than an identical one.

(ii) That *g*_21_ < 0 ∧ *g*_12_ < 0. Thus the sign of Eq. (4) is simultaneously determined by an isolated effect (the first term) and a coupling effect (the second term). To guarantee ${\rm{\Delta }}E > 0$, we derive

5 $$\begin{array}{l}c+{x}_{{\rm{f1}}} < -\frac{c({g}_{21}+{g}_{12})}{{g}_{11}}\mathrm{.}\end{array}$$

A simple example is an undirected chain with two nodes in which the adjacency matrix elements are *a*_12_ = *a*_21_ = 1 and *a*_11_ = *a*_22_ = −1.25 (see Method for details). For simplicity, we set *c* = 1 and simplify Eq. (5) to be *x*_f1_ < 0.6. Selecting *x*_f1_ = 0.5 and substituting these parameters into Eq. (4), we obtain Δ*E* = 0.125. Although it is nearly impossible to define the condition of Δ*E* in networks with complicated topological structures, the competition between two kinds of term allows us to conclude that ${E}_{{\rm{NI}}} > {E}_{{\rm{ID}}}$ (${\rm{\Delta }}E > 0$).

**Figure 4:**
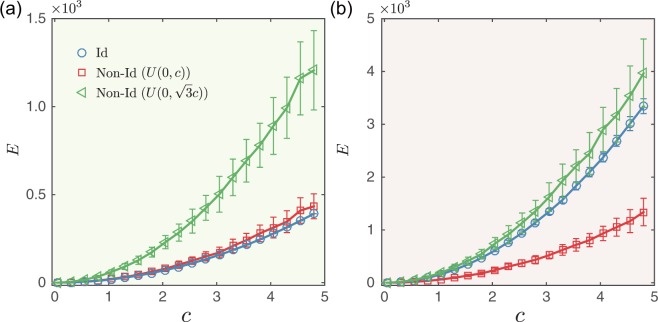
Control energy of directing the real networks to identical (Id) and non-identical (Non-Id) final state modes. Control energy *E* as a function of distance *c* for (**a**) Zachary karate club network and (**b**) Seagrass food web network. The error bars represent standard deviations and each data point is an average over 100 independent realizations.

To verify that these synthetic network findings occur in real-world systems, we investigate the control energy required to drive an undirected network (the Zachary Karate Club) and a directed network (the Seagrass Food Web) towards non-identical and identical final states in Fig. 4. Although the results are consistent with those in modeled networks, the energy gap Δ*E* is narrower.

### Conformity-based model

We now examine a more complicated and realistic model that can capture the dynamics among individuals as they achieve a globally identical final state. We incorporate conformity behavior, i.e., each node tends to follow the state predominating in its neighborhood. Thus the state of individual *i* at time *t* + 1 is

6 $${x}_{i}(t+\mathrm{1)}=\sum _{j=1}^{{k}_{i}}{x}_{j}(t)/{k}_{i},$$

where *x*_*j*_(*t*) is the state of node *i*’s neighbor *j* at time *t* and ${k}_{i}={\sum }_{j=1}^{N}{A}_{ij}$ is the degree of node *i*. Equation (6) indicates that an individual’s state at the next time step *t* + 1 is the average state of its neighbors at the current step *t*. We extend Eq. (6) to the networked system, and the dynamics of the conformity behavior of *N* nodes are

7 $${\bf{x}}(t+\mathrm{1)}={K}^{-1}A{\bf{x}}(t)+B{\bf{u}}(t),$$

where *K*^−1^ is the diagonal matrix of the inverse of the node degrees that captures the overall conformity behavior. The system described by Eq. (7) remains linear. Though the controllability framework of the discrete-time system is similar to that in a continuous-time system^42^, the final time *t*_f_ should be larger than or equal to *N* − 1 to guarantee the controllability Gramian matrix of discrete-time system is invertible (we choose *t*_f_ → ∞). Note that control energy *E* is simultaneously determined by the matrix *K*^−1^ and the coupling matrix *A* of the system, which allows the control energy to differ from that when conformity is absent.

**Figure 5:**
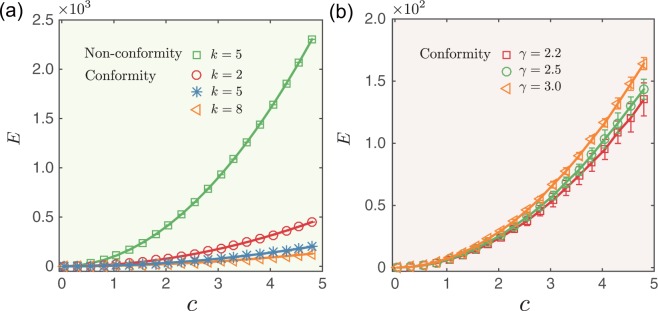
Control energy of directing the undirected random network with conformity dynamics to identical final state. Control energy *E* as a function of distance *c* for (**a**) random networks and (**b**) scale-free networks. Average degree *k* = 5 and network size *N* = 200. Since the control energy for scale-free network without conformity is the same as that for the random network shown in (**a**), it is not presented in (**b**). The error bars represent standard deviations and each data point is an average over 100 independent realizations.

Figure 5 shows the control energy *E* required for a conformity-based dynamical system to achieve an identical final state. We find that conformity behavior facilitates the control energy of random networks towards identical final states. Because conformity is strongly encouraged in dense networks, the required control energy *E* is lower in networks with a larger average degree or in networks that are heterogeneous.

### Optimal control strategy

To determine the optimal driver nodes set for minimizing the control energy, we note that the control energy *E* decays as the length of the longest path from external inputs becomes shorter^30^. To achieve an identical final state, we use a multi-chain network and compare the control energy of different selection strategies applied to the driver nodes set.

**Figure 6:**
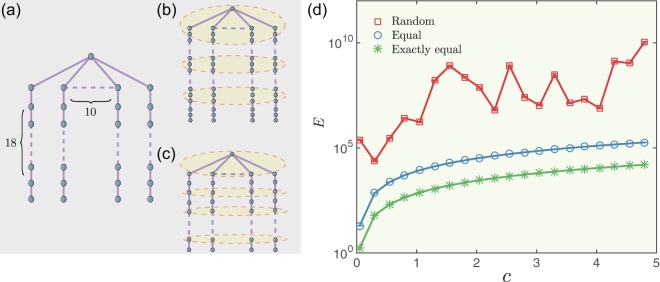
Optimal selection of driver nodes with lower control energy. (**a**) A simple network consisting of 10 chains, and each chain has 18 nodes. (**b**) Equally distributed control strategy. The driver nodes set consists of 3 parts and each part includes 30 driver nodes. (**c**) Exactly equal control strategy. The driver nodes set consists of 9 parts and each part includes 10 driver nodes. The driver nodes in panels (b) and (c) are all marked by yellow. (**d**) Control energy *E* as a function of distance *c* in directing the multi-chain network in three control strategies. The error bars represent standard deviations and each data point is an average over 100 independent realizations.

Figure 6(a) shows a chain-like network (with 10 nodes in each subchain) with *N* = 181 nodes in which the first node of each chain shares the same ancestor. We compare the control energy *E* by using three strategies to select a fraction of *f* = 0.5 nodes as driver nodes, (i) a randomly-distributed set in which the driver nodes are chosen randomly, (ii) an equally-distributed set in which the driver nodes are distributed equally in order to divide the chains into equal segments in a hierarchical structure (see Fig. 6(b)) and (iii) an exactly equal set in which the driver nodes can divide the chains into exactly equal segments in a hierarchical structure (see (Fig. 6(c)). Figure 6(d) shows that the exactly equal set can lead to the minimum control energy, and *E* exponentially grows with the control distance *c*. Thus the key driver nodes for the optimal control are those in a topological position that equally divides the hierarchical structure.

## Discussion

We have combined conformity-based dynamics and complex networks to determine the control energy required to direct a network towards non-identical and identical final states, respectively. In undirected networks, although there is a longer distance between the identical final state and its initial state, the control energy is less than that required to reach a non-identical final state. The critical factor in the role of control energy is the network size, not the network topology. Thus the degree distribution does not significantly affect the level of control energy required to reach an identical final state in either undirected random or scale-free networks. We examine the role of conformity dynamics in directing networks towards an identical final state and find that when there is conformity the control energy is reduced. Using a multi-chain, we find that the driver nodes corresponding to the optimal control strategy are those that divide the hierarchical structure equally.

Our results indicate a possible connection between network control and the mixture of the desired final state. This could shed light on how nodal dynamics and the desired final state affect the control of a complex networked system. We also present an optimal control strategy for energy reduction that suggests a possible direction for future research.

## Methods

The dynamics of an *N*-dimensional linear time-invariant is governed by:

8 $$\dot{{\bf{x}}}(t)=A{\bf{x}}(t)+B{\bf{u}}(t),$$

where *x*(*t*) = [*x*_1_(*t*), *x*_2_(*t*), ..., *x*_*N*_(*t*)]^T^ is the state of the system at time *t*, **u**(*t*) = [*u*_1_(*t*), *u*_2_(*t*), ..., *u*_*M*_(*t*)]^T^ is the external control inputs, and *A* is the adjacency matrix that captures the interaction strength between nodes. *B* is the control matrix that specifies how the inputs are connected to network nodes. Here *B* is a diagonal unit matrix since all nodes are chosen as driver nodes. A dynamical networked system described by Eq. (8) is controllable if a finite number of inputs can steer it from any initial state to any final state within a finite period of time. The driver nodes are the set of nodes driven by external inputs. Given an input **u**(*t*), the corresponding control energy is $E(t)={\int }_{0}^{t}||{\bf{u}}(\tau )|{|}^{2}{\rm{d}}\tau$. Using classical control theory^21^, from the initial state **x**_o_ at time *t* = 0, the minimal energy required to drive the system to any final state **x**_f_ at time *t* = *t*_f_ is

9 $$E({t}_{{\rm{f}}})={{\bf{x}}}_{{\rm{f}}}^{{\rm{T}}}{G}^{-1}({t}_{{\rm{f}}}){{\bf{x}}}_{{\rm{f}}},$$

where $G({t}_{{\rm{f}}})={\int }_{0}^{{t}_{{\rm{f}}}}{{\rm{e}}}^{At}B{B}^{{\rm{T}}}{{\rm{e}}}^{{A}^{{\rm{T}}}t}{\rm{d}}t$ is the symmetric controllability Gramian matrix. Because the control energy decays quickly when the control time *t*_f_ increases, we set *t*_f_ → ∞ and focus on the control energy *E* ≡ *E*(*t*_f_ → ∞)^27^. Here **x**_o_ and **x**_f_ are two vectors with *N* rows for each denoting the initial and final states, respectively. Eq. (9) indicates that the energy *E*(*t*) is determined by both the input signals and the nature of the final state. Prior research has investigated the non-identical final state (NI), but we focus on the minimal energy required to control the system to achieve an identical final state (ID). Here we compare the distinct mixture modes of final state, (i) an identical final state ${{\bf{x}}}_{{\rm{f}}}^{({\rm{ID}})}$, where the state of each node *x*_*i*_(*t*_f_) is a constant *c*, (ii) a non-identical final state ${{\bf{x}}}_{{\rm{f}}}^{({\rm{NI1}})}$, where the state of each node is drawn from the uniform distribution ${x}_{i}({t}_{{\rm{f}}})\sim {\bf{U}}(0,c)$, and (iii) a non-identical final state ${x}_{{\rm{f}}}^{({\rm{NI2}})}$, where the final state follows the uniform distribution ${x}_{i}({t}_{{\rm{f}}})\sim {\bf{U}}(0,\sqrt{3}c)$. Note that we use the constant *c* to adjust the distance between the initial state **x**_o_ and the final state **x**_f_. Following the common convention, we add a self-loop ${A}_{ii}=-\,(\delta +{\sum }_{j=1}^{N}{A}_{ij})$ to each node^27^, where *δ* = 0.25 is a small perturbation that guarantees the stability of the system by which the eigenvalues of the adjacency matrix *A* are all negative. The discrete system may be unstable as *δ* = 0.25, while we can guarantee it is stable by increasing *δ*.
